# Risk management strategies in community-based psychiatry: a narrative review focusing on Italy

**DOI:** 10.1192/j.eurpsy.2026.11714

**Published:** 2026-07-31

**Authors:** G. Listanti, S. De Persis, D. Tripi, S. Ferracuti

**Affiliations:** 1 Department of Human Neuroscience, Sapienza, University of Rome, Roma; 2 Dipartimento Tutela e Promozione Salute Mentale, Azienda Sanitaria Locale, Rieti, Italy

## Abstract

**Introduction:**

In psychiatry, Risk Management (RM) has mainly focused on hospital settings, while incidents of violence and burnout are increasingly affecting community-based services. In Italy, after the reform of forensic psychiatry, local mental health departments are fully responsible for the treatment of psychiatric offenders. The Italian National Action Plan for Mental Health 2025–2030 now formally recognizes RM as a strategic priority.

**Objectives:**

This study provides an overview of key domains, critical issues, objectives and intervention strategies for RM in Italian community-based mental health services.

**Methods:**

We conducted a non-systematic review of international and Italian literature.

**Results:**

Our results identify 5 key domains of clinical risk:

- Staff well-being and safety
- Hospital-community continuity
- Forensic patients management
- Informed consent
- Training and guidelines

Specific critical issues, objectives and intervention strategies for each risk domain are summarized in Table 1.

**Table 1.** Critical issues, objectives and intervention strategies for each clinical risk domain in community-based psychiatry. Table 1. Long description.

Risk domain	Critical Issues	Objectives	Intervention Strategies
**Staff well-being & safety**	High emotional load; staff shortages; unsafe home visits; risk of aggressive behaviours	Burnout & violence prevention; implementing reporting systems	Person-centred culture; pre-visit risk assessment & team visits; de-escalation/crisis training
**Hospital-community continuity**	Limited service access; geographic & organizational barriers; chronic disorders	Strengthen transition pathways; reduce access barriers	Family engagement; proactive post-discharge follow-up; strengthening inter-service links
**Forensic patients management**	High clinical complexity & aggression; difficult care/containment balance; poly-drug treatments	Structured recidivism risk assessment; develop specialized teams & pathways	Use of standardized tools; joint DSM-REMS project development; specialized staff training
**Informed consent**	Fluctuating decisional capacity; clinical judgment-only assessment; poor SDM adoption	Regular capacity assessment; promote SDM & recovery	Use of standardized tools; recovery-oriented practices; promote SDM in rehab projects; family engagement
**Training & Guidelines**	Evidence-practice gap; heterogeneous training; difficulty implementing procedures & monitoring safety outcomes	Continuous EBP training & safety indicators	Continuous staff education on EBP implementation & safety outcome monitoring

**Conclusions:**

RM in community-based psychiatry in Italy requires a systemic approach. It is essential that Italian mental health departments invest their resources in trained personnel, standardized tools, evidence-based practices, and a culture of shared decision-making. As a non-systematic review, this study is not exhaustive and further research is needed to fully map RM practices in Italian community-based psychiatric services.

**Disclosure of Interest:**

None Declared

## Long descriptions

### Table 1. Long description

The table is organized into four columns: Risk domain, Critical Issues, Objectives, and Intervention Strategies.

* Staff well-being and safety: Critical issues include high emotional load, staff shortages, unsafe home visits, and risk of aggressive behaviors. Objectives focus on burnout and violence prevention and reporting systems. Strategies include person-centered culture, pre-visit risk assessments, team visits, and de-escalation training.

* Hospital-community continuity: Critical issues include limited service access, geographic and organizational barriers, and chronic disorders. Objectives focus on strengthening transition pathways and reducing access barriers. Strategies include family engagement, proactive post-discharge follow-up, and strengthening inter-service links.

* Forensic patients management: Critical issues include high clinical complexity, aggression, care-containment balance, and poly-drug treatments. Objectives focus on structured recidivism risk assessment and specialized teams. Strategies include standardized tools, joint D S M-R E M S project development, and specialized staff training.

* Informed consent: Critical issues include fluctuating decisional capacity, clinical judgment-only assessment, and poor S D M adoption. Objectives focus on regular capacity assessment and promoting S D M and recovery. Strategies include standardized tools, recovery-oriented practices, and family engagement.

* Training and Guidelines: Critical issues include evidence-practice gaps, heterogeneous training, and difficulty monitoring safety outcomes. Objectives focus on continuous E B P training and safety indicators. Strategies include continuous staff education on E B P implementation and safety outcome monitoring.

Navigate back to Table 1..

